# Behavioral Effects of a Potential Novel TAAR1 Antagonist

**DOI:** 10.3389/fphar.2018.00953

**Published:** 2018-09-04

**Authors:** Vincent M. Lam, Catharine A. Mielnik, Corey Baimel, Pieter Beerepoot, Stefano Espinoza, Ilya Sukhanov, Wendy Horsfall, Raul R. Gainetdinov, Stephanie L. Borgland, Amy J. Ramsey, Ali Salahpour

**Affiliations:** ^1^Department of Pharmacology and Toxicology, University of Toronto, Toronto, ON, Canada; ^2^Department of Physiology and Pharmacology, University of Calgary, Calgary, AB, Canada; ^3^Boston Children’s Hospital, F.M. Kirby Center for Neurobiology, Harvard Medical School, Boston, MA, United States; ^4^Department of Neuroscience and Brain Technologies, Fondazione Istituto Italiano di Tecnologia, Genoa, Italy; ^5^Pavlov First Saint Petersburg State Medical University, Valdman Institute of Pharmacology, Saint Petersburg, Russia; ^6^Institute of Translational Biomedicine, Saint Petersburg State University, Saint Petersburg, Russia

**Keywords:** TAAR1, dopamine transporter (DAT), cocaine, amphetamine, locomotor activity, electrophysiology

## Abstract

The trace amine associated receptor 1 (TAAR1) is a G-protein coupled receptor expressed in the monoaminergic regions of the brain, and represents a potential novel therapeutic target for the treatment of neurological disorders. While selective agonists for TAAR1 have been successfully identified, only one high affinity TAAR1 antagonist has been described thus far. We previously identified four potential low potency TAAR1 antagonists through an *in silico* screen on a TAAR1 homology model. One of the identified antagonists (compound **22**) was predicted to have favorable physicochemical properties, which would allow the drug to cross the blood brain barrier. *In vivo* studies were therefore carried out and showed that compound **22** potentiates amphetamine- and cocaine-mediated locomotor activity. Furthermore, electrophysiology experiments demonstrated that compound **22** increased firing of dopamine neurons similar to EPPTB, the only known TAAR1 antagonist. In order to assess whether the effects of compound **22** were mediated through TAAR1, experiments were carried out on TAAR1-KO mice. The results showed that compound **22** is able to enhance amphetamine- and cocaine-mediated locomotor activity, even in TAAR1-KO mice, suggesting that the *in vivo* effects of this compound are not mediated by TAAR1. In collaboration with Psychoactive Drug Screening Program, we attempted to determine the targets for compound **22**. Psychoactive Drug Screening Program (PDSP) results suggested several potential targets for compound **22** including, the dopamine, norepinephrine and serotonin transporters; as well as sigma 1 and 2 receptors. Our follow-up studies using heterologous cell systems showed that the dopamine transporter is not a target of compound **22**. Therefore, the biological target of compound **22** mediating its psychoactive effects still remains unknown.

## Introduction

The trace amine associated receptor 1 (TAAR1) is a GPCR that in part acts as an autoreceptor in presynaptic monoamine neurons, where TAAR1 signaling decreases the firing rate of dopaminergic neurons and dopamine release from terminals (Bradaia et al., 2009; Revel et al., 2011; Leo et al., 2014; Lam et al., 2015a). In addition, TAAR1 has also been shown to interact with the dopamine D2 receptors both pre- and post-synaptically (Leo et al., 2014; Espinoza et al., 2015); as well as in heterologous cell systems (Espinoza et al., 2011). Indeed, functionally it has been proposed that the TAAR1-D2 heteromer negatively modulates GSK3β signaling (Harmeier et al., 2015). Due to these mechanisms of TAAR1 action, there has been much focus on TAAR1 as a potential target for the treatment of neurological and psychiatric diseases, which can arise from the dysregulation of the brain dopamine system.

Selective TAAR1 agonists based on either the 2-benzyl-imidazoline (Galley et al., 2012) or 2-aminooxazole backbones (Galley et al., 2016) have been shown to decrease the firing rate of dopaminergic neurons. These observations led to the testing of TAAR1 agonists as potential treatments for schizophrenia, a disease characterized by *hyper*-dopaminergia (Brisch et al., 2014). In a series of recent studies, TAAR1 agonists demonstrated antipsychotic activity (Revel et al., 2011, 2012, 2013) in the animal models of schizophrenia. Interestingly, TAAR1 agonists are shown to have similar efficacy in improving both positive and negative symptoms of schizophrenia and also were able to improve few cognitive deficits. Moreover, RO5263397, a partial TAAR1 agonist, does not have the same adverse metabolic side effects as olanzapine, and co-treatment of RO5263397 with olanzapine reduced the metabolic side effects observed with olanzapine alone (Revel et al., 2013). Although still no TAAR1 ligand has been approved for clinical use, TAAR1 remains an intriguing and novel drug target for schizophrenia.

In disease states of *hypo*-dopaminergic dysregulation such as Parkinson’s disease, a recent study has shown that TAAR1 signaling is also involved and that TAAR1 antagonism could potentially slow the progression of the disease (Alvarsson et al., 2015). Unlike TAAR1 agonists which display antipsychotic activity, a TAAR1 antagonist should enhance dopamine signaling and be useful for the treatment of diseases arising from *hypo*-dopaminergia such as Parkinsons’s disease. In contrast to the several selective synthetic TAAR1 agonists available, there only exists one selective high affinity TAAR1 antagonist, EPPTB (Bradaia et al., 2009). Unlike TAAR1 agonists, the potential for TAAR1 antagonists in the treatment of disorders arising from *hypo*-dopaminergia has not been explored *in vivo*; due to poor *in vivo* pharmacokinetic properties of EPPTB (Stalder et al., 2011). To identify novel TAAR1 antagonists, we have recently used *in silico* screening of commercially available compounds on a TAAR1 homology model (Cichero et al., 2014; Lam et al., 2015b). These studies allowed for the identification of low affinity TAAR1 antagonists, which were validated *in vitro*. In the present study, the behavioral characterization of a previously discovered novel antagonist (compound **22**) was performed *in vivo*. Compound **22** is predicted to have good pharmacokinetic properties that allow the drug to cross the blood brain barrier (BBB). Our data indicated that this compound is able to regulate dopamine transmission by potentiating the locomotor stimulating effects of the psychostimulants cocaine and amphetamine, however, these effects are found to be independent of TAAR1.

## Materials and Methods

Cocaine hydrochloride (Medisca, New York, NY; Batch: 0723-06) and amphetamine (Tocris Bioscience, Bristol, United Kingdom; Batch: 4A/137502) were handled and stored according to regulations set by Health Canada. Compound **22** was purchased from Enamine Ltd. (Kiev, Ukraine). Cell culture reagents and buffers were obtained from Sigma-Aldrich (St. Louis, MO, United States) and Life Technologies (Carlsbad, CA, United States). HEK293 (CRL-1573) cells were purchased from American Type Culture Collection (Hopkinton, United States). Poly-D-lysine was purchased from Sigma-Aldrich and prepared by dissolving the powder to a concentration of 1 mg/mL in ddH2O. Polyethylenimine (PEI) was purchased from Polyscience Inc. (Warminster, PA, United States) and dissolved to a concentration of 1 mg/mL. Aliquots of PEI were stored at -80°C.

The human HA-DAT construct was provided by Sorkina et al. (2006). The backbone of this construct is the peYFP-c1 vector, where the YFP is located on the N-terminus of DAT. In addition, an HA epitope was added onto the second extracellular loop replacing residues 193–203.

All animals were housed in the Division of Comparative Medicine at the University of Toronto. Procedures were conducted in accordance with the Canadian Council for Animal Care and the University of Toronto Faculty of Medicine and Pharmacy Animal Care Committee. Mice were housed 1–4 per cage with 12 h light/dark cycles (7:00–19:00), with *ad libitum* access to food (Teklad, Envigo, IN, United States) and water.

### Cell Culture

HEK293 cells were cultured in Dulbecco’s Modified Eagle Serum (DMEM), supplemented with 10% fetal bovine serum (Sigma-Aldrich), and maintained at 37°C with 5% CO_2_ in a humidified atmosphere. Cells were passaged 24 h prior to transfection at 50% confluency (∼2 × 10^6^ cells in a 10 cm plate). Transfections were carried out using the PEI method as described previously (Ehrhardt et al., 2006; Lam et al., 2013; Beerepoot et al., 2016). PEI and plasmid DNA (3 μl:1 μg PEI:DNA ratio) were added into separate tubes (tube 1: PEI, tube 2: DNA) followed by 200 μL of DMEM into each tube, containing no supplements. Tubes were allowed to incubate for 5 min before the two tubes were combined (PEI with DNA). The PEI:DNA mixture was then further incubated for 30 min at room temperature and subsequently added drop wise to a 10 cm plate containing HEK293 cells at 50% confluency. For stable cell line generation with the HA-DAT construct, 24 h after transfection, media was replaced with selection media containing G418 (500 μg/mL, Bioshop, Burlington, ON, Canada). Clonal cell lines were generated by picking individual colonies ∼2 weeks post-transfection. Expression was confirmed by western blot and fluorescence microscopy.

### Fluorescent Dopamine Uptake Assay

Fluorescent dopamine uptake assay kits were purchased from Molecular Devices (Sunnyvale, CA; catalog #: R6138). Stable cells expressing human HA-DAT were seeded on poly-D-lysine treated, black clear-bottom 96-well plates (Corning Catalog #: 3603) at a density of 1 × 10^5^ cells/well, and incubated for 24 h prior to the start of the uptake experiment. The media was removed and replaced with 80 μL of assay buffer (20 mM HEPES, 1× HBSS, pH 7.4), followed by 10 μL of either 2× concentrated compound **22**, 10 μL of 2× concentrated cocaine, or vehicle solutions, previously dissolved in assay buffer. The plates were then incubated for 30 min at 37°C. Following incubation, 100 μL of dye solution was added and fluorescence intensity was measured for 30 min at 37°C using the SpectraMax M3 (Molecular Devices, excitation: 440 nm, emission: 520 nm). The rate of reaction (slope of the curve in the linear range) was taken as the readout for the assay.

### Electrophysiology

All protocols were in accordance with the ethical guidelines established by the Canadian Council for Animal Care and were approved by the University of Calgary Animal Care Committees. All mice were housed in groups of 2–5 and were maintained on a 12-h light: dark schedule and were given food and water *ad libitum*. Experiments were performed during the animal’s light cycle.

All electrophysiological recordings were performed in slice preparations from C57Bl/6J mice (P21-P30). Briefly, mice were anaesthetized with isoflurane and transcardially perfused with an ice-cold sucrose solution containing (in mM): 50 sucrose, 26.2 NaHCO3, 1.25 glucose, 4.9 MgCl2, 3 kynurenic acid, 0.1 CaCl2, and 1.32 ascorbic acid in bicarbonate-buffered solution (aCSF, described below). Mice were then decapitated and brains were extracted. Horizontal sections (180 μm) containing the VTA were cut on a vibratome (Leica, Nussloch, Germany) and incubated in a holding chamber for at least 45 min before being transferred to a recording chamber and superfused with aCSF containing (in mM): 126 NaCl, 1.6 KCl, 1.1 NaH2PO4, 1.4 MgCl2, 2.4 CaCl2, 26 NaHCO3, 11 glucose (32-34oC), and saturated with 95% O2/5% CO2. Cells were visualized on an upright microscope using “Dodt-type” gradient contrast infrared optics (Dodt et al., 2002) and whole-cell recordings were made using a MultiClamp 700B amplifier (Axon Instruments, Union City, CA, United States). Recording electrodes (3–5 MΩ) were filled with (in mM): 136 potassium-D-gluconate, 4 MgCl2, 1.1 HEPES, 5, EGTA, 10 sodium creatine phosphate, 3.4 Mg-ATP, and 0.1 Na2GTP. Putative VTA dopamine neurons were identified by the presence of a large hyperpolarization-activated, cyclic nucleotide-regulated cation (I_h_) current. Spontaneous firing activity was recorded in current-clamp mode. Compound **22** and EPPTB were both dissolved in DMSO and diluted to their final concentration in aCSF and bath applied to slices for 5 min. Firing data for all neurons was analyzed with the MiniAnalysis program (Synaptosoft) using the same criteria. Drug-induced changes in firing are expressed as a percentage of baseline. Drug effects were calculated by comparing the response during the baseline/pre-drug period to the response 5 min after onset of drug administration.

### Experimental Mice

The *Taarl*^-/-^ (TAAR1-KO) mice were obtained from Lundbeck (Wolinsky et al., 2007). All wild type (WT) and TAAR1-KO mice used for experiments were generated from TAAR1-KO heterozygous mice in a C57BL/6J x 129S1/Sv mixed background.

### Behavioral Experiments

Experimentally naïve mice, of at least 12 weeks of age, were used for all behavioral experiments. The mice were randomly assigned to treatment or control groups, balanced by sex and weight. Locomotor activity was assessed using the automated locomotor analysis monitors (Omnitech Electronics, Columbus, OH, United States). The apparatus included four open field monitors. Each Open Field monitor consisted of sets of 16 light beams arrayed in the horizontal X and Y axes. The hardware detected beams broken by the animal, which allowed the software to determine the location of the mouse in the cage. Total distance covered by mice was used to characterize locomotor activity of the animals. The monitors were divided into four compartments (20 cm × 20 cm). Animals were tested individually for defined periods with 5-min intervals. The mice were first weighed and then placed into the apparatus, allowing for 30 min habituation. Following the habituation, the mice were removed from the al apparatus and injected with drugs (see below for administration) or vehicle and returned immediately to the locomotor chamber. The locomotor activity was then measured for additional 1 h. After the experiments, the animals were euthanized by cervical dislocation.

### Drug Administration

In all behavioral studies, compound **22** was co-injected with saline, cocaine or amphetamine. All drug solutions were prepared freshly on the day of the experiment and injected i.p. at the volume of 10 ml/kg. Cocaine hydrochloride was dissolved in 0.9% saline at a concentration of 1 mg/mL. Amphetamine was dissolved in 0.9% saline at a concentration of 0.2 mg/mL. Compound **22** was then dissolved into the cocaine or amphetamine solutions, respectively, to the correct dose for the experiment.

### Statistical Analysis

Data analyses were performed with Graphpad Prism 5.01 (GraphPad Software, Inc., La Jolla, CA). Linear regression analysis was used to quantify fluorescent dopamine transporter uptake activity. Dose response curves were fitted with non-linear curve fitting. Two-tailed Student’s *t*-tests or one-way ANOVA analysis with Dunnett’s *post hoc* correction was used where appropriate to determine differences between data sets.

## Results

### Predicted Chemical Properties

Previously, we discovered four potential low potency antagonists of TAAR1 (Lam et al., 2015b). These four identified compounds were assessed for their potential suitability for *in vivo* use, along with their ability to cross the blood brain barrier. To assess a compound’s permeability of BBB, we followed the criteria outlined by an extensive review of marketed drugs for CNS targets, which yielded a series of chemical properties that could predict BBB penetration (Pajouhesh and Lenz, 2005). The following six criteria were used to evaluate our compounds: (1) liquid water partition coefficient (logP), (2) total polar surface area (tPSA), (3) hydrogen bond donor, (4) hydrogen bond acceptor, (5) rotatable bonds, and (6) molecular weight (Pajouhesh and Lenz, 2005). The physical properties of the antagonist hits from Lam et al. (2015b) (compound **9**, **16**, **22**, and **24**) were estimated (**Table 1**). These four compounds shared similar chemical properties, with the largest differences seen in the logP and tPSA. Based on these predicted values, compound **22** and **24** had the most favorable logP values at 3.30 and 2.98, respectively, whereas compound **9** and **16** had logP values of 1.95 and 1.18, respectively. Therefore, compound **22** and **24** had superior predicted chemical properties for crossing the blood brain barrier. However, due to the constraints of commercial availability, compound **22** was chosen for use in the *in vivo* studies described here.

**Table 1:** Comparison of predicted physical properties of antagonist hits from the PubChem database (Wang et al., 2012).

Cpd	2D structure	LogP^a^	H-Bond Donor	H-Bond Acceptor	tPSA(A^2^)^b^	M.W. (g/mol)	Rotatable Bonds
9	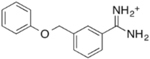	1.95	4	3	61	227.29	4

16	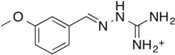	1.18	5	5	85	193.23	4

22	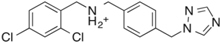	3.3	2	4	47	348.26	6

24	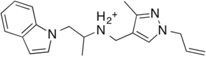	2.98	2	4	39	309.43	7


*^a^Predicted using xlogP (Cheng et al., 2007). ^b^topilogical polar surface area (tPSA).*

### Behavioral Experiments in WT C57BL/6J and TAAR1-KO Mice

It has been previously shown that the TAAR1-KO mice have a potentiated response to the psychostimulant locomotor inducing effects of amphetamine and cocaine (Wolinsky et al., 2007; Lindemann et al., 2008; Di Cara et al., 2011). Therefore, we used locomotor activity as our *in vivo* readout for the testing of compound **22**. We hypothesized that a functional TAAR1 antagonist in WT mice should mimic the phenotypes that are seen in the TAAR1-KO mice, and potentiate their locomotor response to amphetamine and cocaine. Behavioral experiments with compound **22** were carried out in C57BL/6J mice, as well as in TAAR1-KO mice.

### Compound 22 Effects on Basal Locomotor Activity in C57BL/6J Mice

**FIGURE 1:**
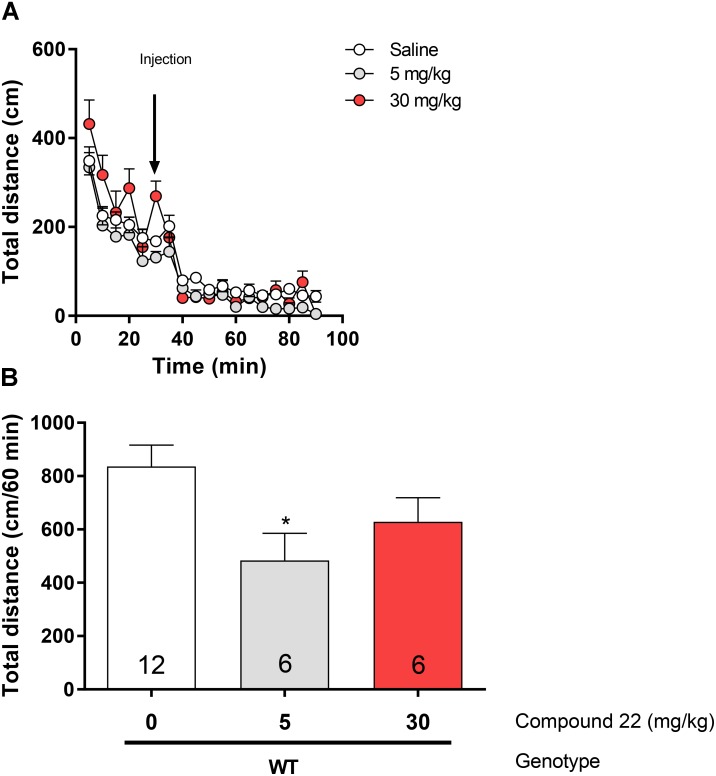
*In vivo* studies with compound **22** on basal locomotor activity. Wild type C57BL/6J mice were first habituated for 30 min followed by co-injection of saline or compound **22** at a dose of 5 or 30 mg/kg. The locomotor activity was assessed for 60 min after injection. **(A)** Locomotor activity over time for saline only or co-injected with 5 or 30 mg/kg compound **22**. **(B)** Sum of locomotor activity over 60 min after the injection of saline or compound **22**. Data are means ± SEM; *N* = 6 for compound **22** treated alone and *N* = 12 for saline treated mice. One-way ANOVA was performed [*F*(3, 32) = 3.49, *p* = 0.027] followed by Dunnett’s *post hoc* analyses (^∗^*p* < 0.05). Data are means ± SEM.

In order to assess the effects of compound **22** on basal locomotor activity, C57BL/6 mice were injected with doses of 5 and 30 mg/kg of compound **22** or vehicle (**Figure 1**). At the dose of 5 mg/kg, compound **22** inhibited basal locomotor activity by 58% (^∗^*p* = 0.02). Although not significant, there was a trend toward a decrease in locomotor activity at the dose of 30 mg/kg as well (26% decrease, *p* = 0.15). Based on these results, compound **22**, when administered alone, did not stimulate locomotor activity.

### Amphetamine Co-injection With Compound 22 in C57BL/6J Mice

**FIGURE 2:**
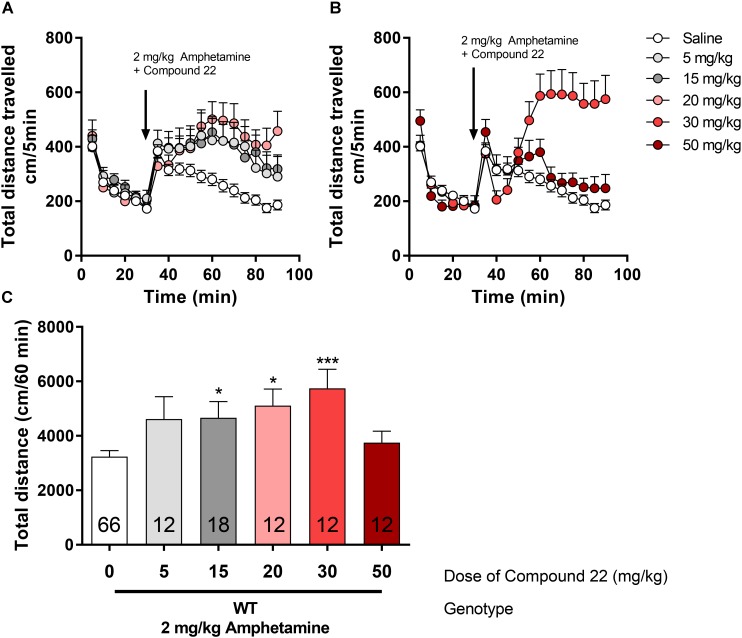
*In vivo* studies with compound **22** co-injected with amphetamine in WT mice. Wild type C57BL/6J mice were first habituated for 30 min followed by the co-injection of amphetamine (2 mg/kg) and saline or compound **22** at doses 5, 15, 20, 25, 30, and 50 mg/kg. The locomotor activity was assessed for 60 min after the injection. **(A)** Locomotor activity over time for 2 mg/kg amphetamine only or co-injected with 5, 15, or 20 mg/kg compound **22**; **(B)** locomotor activity over time for 2 mg/kg amphetamine only or co-injected with 30 or 50 mg/kg compound **22**; **(C)** sum of locomotor activity over 60 min after the injection of amphetamine and compound **22**. Data are means ± SEM; *N* = 12–18 for compound **22** treated alone and *N* = 66 for amphetamine treated alone. A one way ANOVA was performed [*F*(5,126) = 4.788, *p* = 0.0005] followed by Dunnett’s *post hoc* analyses (^∗^*p* < 0.05, ^∗∗∗^*p* < 0.001).

The effect of compound **22** (5, 15, 20, 30, and 50 mg/kg) on locomotor activity, in mice, in combination to a single, sub-maximal dose of amphetamine (2 mg/kg) was carried out (**Figure 2**). Treatment of WT C57BL/6J mice with 15 mg/kg of compound **22** showed enhanced amphetamine locomotor response by 44% (^∗^*p* = 0.04). At doses of 20 or 30 mg/kg of compound **22**, mice exhibited 57% (^∗^*p* = 0.02) and 77% (^∗∗∗^*p* = 0.0009) increases in amphetamine-stimulate motor activity, respectively. Although not significant, there was a trend toward an increase in locomotor activity at the dose of 5 mg/kg as well (28% increase, *p* = 0.32). There was no difference in locomotor activity at the dose of 50 mg/kg of compound **22**.

### Cocaine Co-injection With Compound 22 in C57BL/6J Mice

**FIGURE 3:**
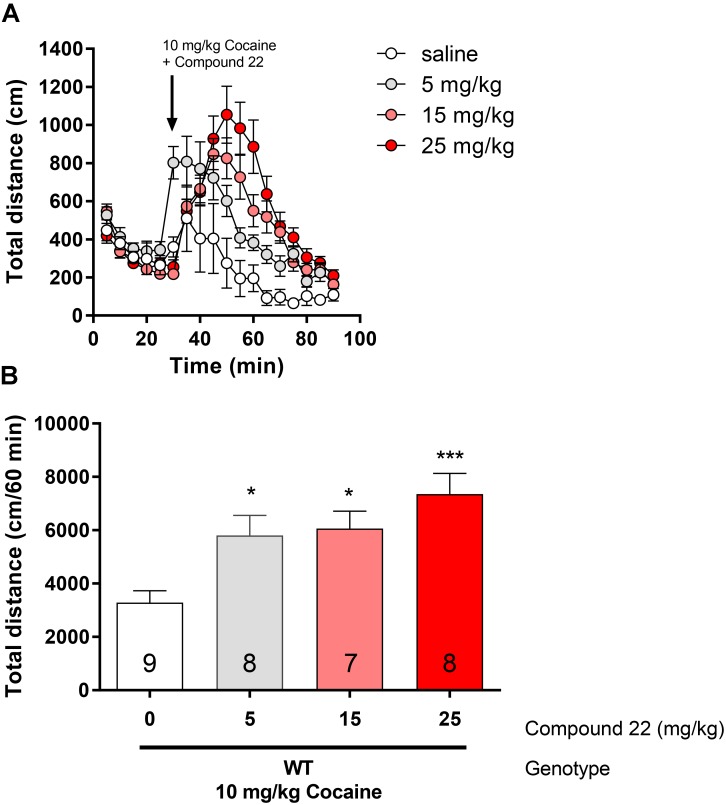
*In vivo* studies with compound **22** co-injected with cocaine. Wild type C57BL/6J mice were first habituated for 30 min followed by co-injection of cocaine (10 mg/kg) and saline or compound **22** at doses 5, 15, and 25 mg/kg. The locomotor activity was assessed for 60 min after the injection. **(A)** Locomotor activity over time for 10 mg/kg cocaine only or co-injected with 5, 15, or 25 mg/kg compound **22**; **(B)** sum of locomotor activity over 60 min after injection of cocaine and compound **22**. Data are means ± SEM; *N* = 7–9. One way ANOVA was performed [*F*(3,28) = 7.138, *p* = 0.001] followed by Dunnett’s *post hoc* analyses (^∗^*p* < 0.05, ^∗∗∗^*p* < 0.001).

Since compound **22** enhanced amphetamine-induced locomotor response, we assessed if compound **22** could also enhance cocaine-induced locomotion in WT C57BL/6J mice (**Figure 3**). Using a single sub-maximal dose of cocaine (10 mg/kg, Orsini et al., 2005), three doses of compound **22** were tested (5, 15, and 25 mg/kg). 5 and 15 mg/kg of compound **22** increased cocaine locomotor activity by 77% (^∗^*p* = 0.03) and 84% (^∗^*p* = 0.02), respectively. At a dose of 25 mg/kg of compound **22**, the mice had a 124% (^∗∗∗^*p* = 0.003) increase in cocaine-induced locomotor activity, clearly indicating that compound **22** enhanced cocaine-induced locomotor activity.

### Effects of Compound 22 Co-injection With Amphetamine and Cocaine on Stereotypic Counts in C57BL/6J Mice

When compound **22** was co-administered with amphetamine we found that there is a statistically significant increase in stereotypic counts at doses of 5 and 20 mg/kg of compound **22** (**Supplementary Figure 1A**). Furthermore, when compound was co-injected with cocaine, there was an increase in stereotypic counts at doses of 5 and 25 mg/kg of compound **22** (**Supplementary Figure 1B**). These results indicate that in addition to locomotor activity, compound **22** can enhance stereotypic counts.

### Effect of Compound 22 on Firing of Dopamine Neurons

**FIGURE 4:**
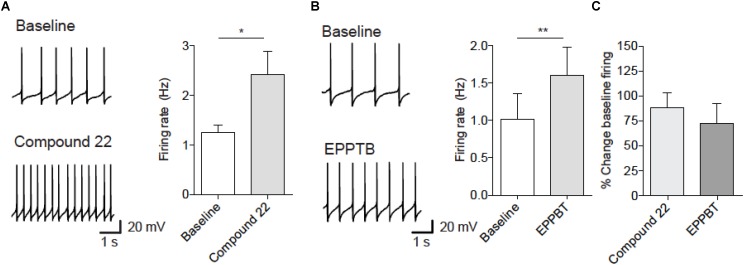
Compound **22** and EPPTB increase spontaneous firing of VTA dopamine neurons. **(A)** Left, example traces of firing before (upper) or after compound **22** (lower). Right, firing rate significantly increased 5 min after onset of bath application of compound **22** (100 μM) [paired *t*-test; *t*(4) = 3.67, ^∗^*p* = 0.02, *N* = 5]. **(B)** Left, example traces of firing before (upper) or after EPPTB (lower). Right, firing rate significantly increased 5 min after onset of bath application of EPPTB (10 nM) [paired *t*-test; *t*(3) = 8.74, ^∗∗^*p* = 0.003, *N* = 4]. **(C)** Effect size of compound **22** or EPPTB. Bars are mean ± SEM.

Previous studies have shown that EPPTB increases the firing rate of dopamine neurons (Bradaia et al., 2009; De Gregorio et al., 2016). We therefore assessed the effects of compound **22** on firing rate of dopamine neurons. At a dose of 100 μM, compound **22** increased the firing rate of dopaminergic neurons from VTA by 88% (**Figure 4**). As a positive control we show that EPPTB caused a 74% increase in firing of dopamine neurons at a dose of 10 nM (**Figure 4**). These observations showing that compound **22** increases the firing rate of dopamine neurons could partially explain its ability to enhance amphetamine and cocaine locomotor stimulating effects.

### Compound 22 Effects on Basal Locomotor Activity in TAAR1-KO Mice

**FIGURE 5:**
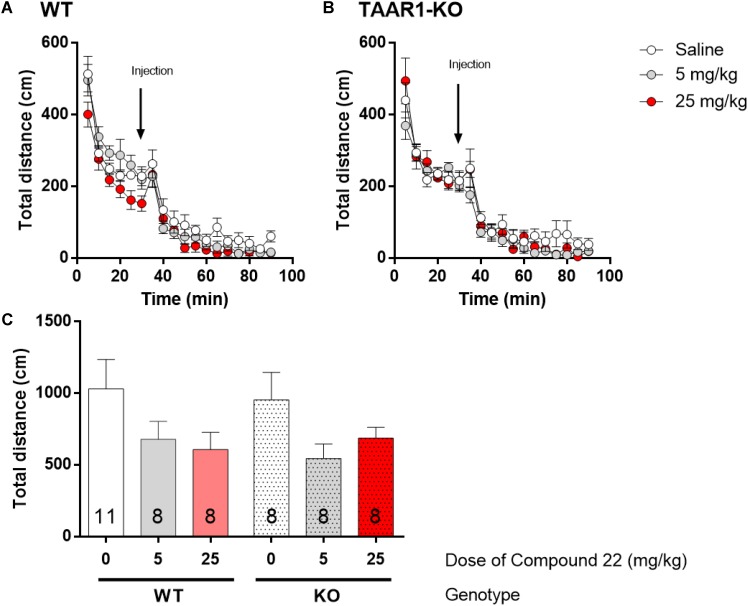
*In vivo* studies with TAAR1-KO mice and compound **22** on basal locomotor activity. TAAR1-KO mice and WT littermates (C57BL/6J × 129S2/Sv) were first habituated for 30 min followed by saline or compound **22** (5 and 25 mg/kg). The locomotor activity was assessed for 60 min following the injection. **(A)** WT locomotor activity over time for saline or compound **22** (5 and 25 mg/kg); **(B)** TAAR1-KO locomotor activity over time for saline only or compound **22** (5 and 25 mg/kg); **(C)** sum of locomotor activity over 60 min after injection of compound **22** in WT (solid bars) or TAAR1-KO mice (dotted bars). Data are means ± SEM; *N* = 8–11. One-way ANOVA was performed for each genotype; WT [*F*(2,24) = 1.915, *p* = 0.1691] and TAAR1-KO [*F*(2,21) = 2.431, *p* = 0.1123] followed by Dunnett’s *post hoc* analyses.

To investigate the response of the TAAR1-KO mice to compound **22**, the locomotor activity of TAAR1-KO and WT littermates were tested with different doses of compound **22** alone. Previous experiments in C57BL/6J mice indicated that compound **22** did not stimulate basal locomotor activity. Two doses of compound **22** (5 and 25 mg/kg) were used in TAAR1-KO mice or their WT littermates (**Figure 5**). For both doses tested, compound **22** did not significantly alter the basal locomotor activity of the TAAR1-KO mice or their WT littermates.

### Amphetamine Co-injection with Compound 22 in the TAAR1-KO Mice

**FIGURE 6:**
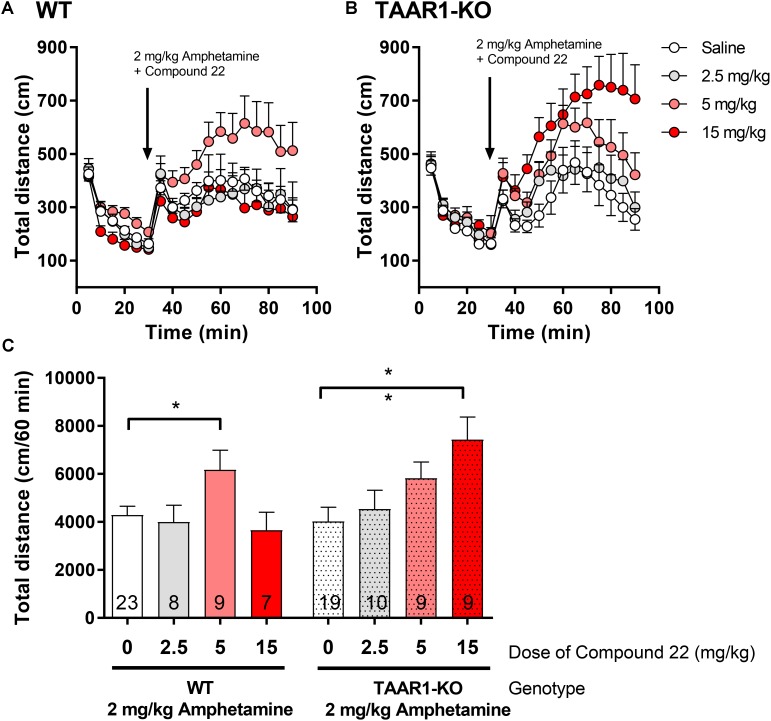
*In vivo* studies with TAAR1-KO mice co-injected with compound **22** and amphetamine. TAAR1-KO mice and their WT littermates (C57BL/6J × 129S2/Sv) were first habituated for 30 min followed by co-injection of amphetamine (2 mg/kg) and saline or compound **22** at doses 2.5, 5, and 15 mg/kg. The locomotor activity was assessed for 60 min after the injection. **(A)** WT locomotor activity over time for 2 mg/kg amphetamine only or co-injected with 2.5, 5, and 15 mg/kg of compound **22**; **(B)** TAAR1-KO locomotor activity over time for 2 mg/kg amphetamine only or co-injected with 2.5, 5, and 15 mg/kg of compound **22**; **(C)** sum of locomotor activity over 60 min after the injection of amphetamine and compound **22** in WT (solid bars) or TAAR1-KO mice (spotted bars). Data are means ± SEM; *N* = 7–23. One-way ANOVA was performed for each genotype; WT [*F*(3,43) = 2.862, *p* = 0.048] and TAAR1-KO [*F*(3,43) = 4.159, *p* = 0.011] followed by Dunnett’s *post hoc* analyses (^∗^*p* < 0.05, ^∗∗^*p* < 0.01).

In order to assess if the *in vivo* effects we observed with compound **22** in WT C57BL/6 mice were due to the antagonism of TAAR1, we repeated our co-injection experiments of compound **22** with amphetamine in TAAR1-KO mice. As with the previous *in vivo* experiments with compound **22**, a single submaximal dose of amphetamine (2 mg/kg) was used. In these experiments, doses of 2.5, 5, and 15 mg/kg of compound **22** were tested in TAAR1-KO mice and their WT littermates. As shown in **Figure 6**, in TAAR1-KO mice, the locomotor stimulating effects of amphetamine were potentiated by 84% with 15 mg/kg of compound **22** (^∗∗^*p* = 0.004). Interestingly, in the WT littermates of TAAR1-KO mice, significant potentiation of amphetamine response was seen at 5 mg/kg (44%, ^∗^*p* = 0.049). Taken together, these results show that enhancement of amphetamine-induced locomotor response by compound **22** is not through a TAAR1 selective mechanism.

### Cocaine Co-injection With Compound 22 in the TAAR1-KO Mice

**FIGURE 7:**
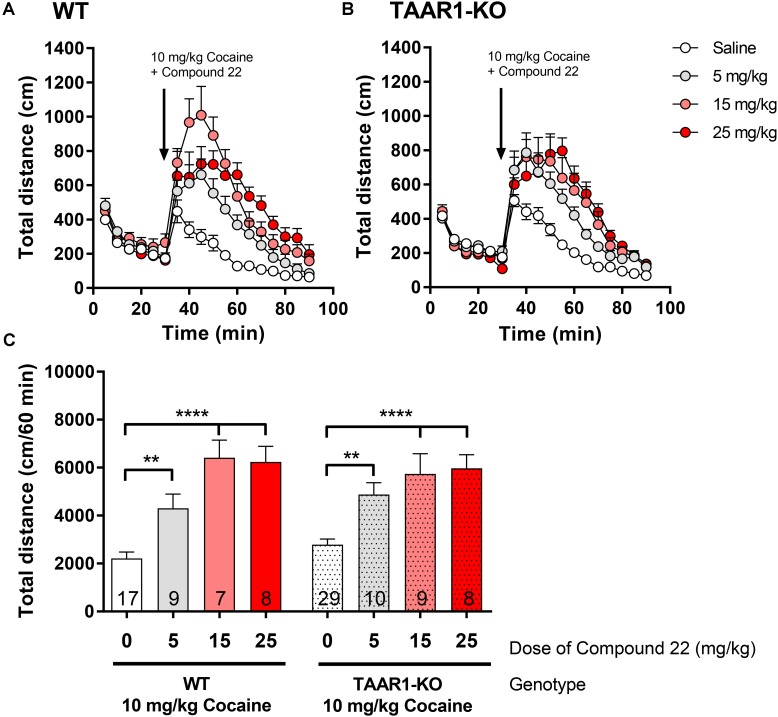
*In vivo* studies with TAAR1-KO mice co-injected with compound **22** and cocaine. TAAR1-KO mice and WT littermates (C57BL/6J × 129S2/Sv) were first habituated for 30 min, followed by co-injection of cocaine (10 mg/kg) with saline or compound **22** (5, 15, and 25 mg/kg). The locomotor activity was assessed for 60 min following the injection. **(A)** WT locomotor activity over time for cocaine (10 mg/kg) only or co-injected with compound **22** (5, 15, and 25 mg/kg), **(B)** TAAR1-KO locomotor activity over time for cocaine (10 mg/kg) only, or co-injected with compound **22** (2.5, 5, and 15 mg/kg). **(C)** Sum of locomotor activity over 60 min following the injection of cocaine and compound **22** in WT (solid bars) or TAAR1-KO mice (dotted bars). Data are means ± SEM; *N* = 7–29. A one-way ANOVA was performed for each genotype; WT [*F*(3,38) = 17.97, *p* < 0.0001] and TAAR1-KO [*F*(3,52) = 13.93, *p* < 0.0001] followed by Dunnett’s *post hoc* analyses (^∗∗^*p* < 0.01, ^∗∗∗∗^*p* < 0.0001).

Since we showed that compound **22** potentiated amphetamine locomotor response did not act through a TAAR1 specific mechanism, next, we investigated whether the same was true for compound **22**-potentiated cocaine locomotor response. Doses of 5, 15, and 25 mg/kg of compound **22** were assessed with cocaine co-injection in the TAAR1-KO mice and their WT littermates. As in previous studies, a submaximal dose of 10 mg/kg of cocaine was used for all locomotor assays (**Figure 7**). All three doses of compound **22** were able to significantly potentiate the locomotor stimulating effects of cocaine in both WT and TAAR1-KO mice. These data further supported that compound **22** is able to potentiate the locomotor stimulant action of amphetamine (**Figure 6**) and cocaine (**Figure 7**) in a TAAR1-independent manner.

### Effects of Compound 22 Co-injection With Amphetamine and Cocaine on Stereotypic Counts in the TAAR1-KO Mice

In the TAAR1-KO mice and their WT littermates, we found a similar trend for increased stereotypic counts compared to locomotor activity. For amphetamine treated mice, the WT animals did not have statistically enhanced stereotypic counts in any dose of compound **22**. In the TAAR1-KO mice, only the dose of 15 mg/kg produced a statistically significant increase in stereotypic counts (**Supplementary Figure 2A**). When compound **22** was co-injected with cocaine, compound **22** enhanced stereotypic counts in WT animals at doses of 15 and 25 mg/kg of compound **22**. Lastly the TAAR1-KO mice showed significant increases in stereotypic counts after cocaine injection for all three doses tested of compound **22** (5, 15, and 25 mg/kg; **Supplementary Figure 2B**).

### Elucidating the Mechanism of Compound 22

Since our data in TAAR1-KO mice indicated that compound **22** modulated the locomotor response to psychostimulants in a TAAR1-independent manner, we next aimed to identify potential targets for compound **22** that would mediate this effect. This was achieved through the identification of potential pharmacological targets of compound **22** using the Psychoactive Drug Screening Program (PDSP) at University of North Carolina-Chapel-Hill. PDSP provides a platform for screening novel psychoactive compounds on human proteins expressed in the central nervous system in order to identify their biological target(s) (Besnard et al., 2012). Binding studies were done on 47 targets at a single dose of compound **22** (10 μM; **Supplementary Table S1**). This primary screen yielded a total of 5 hits for compound **22** (**Table 2**). These hits were the serotonin, dopamine, and norepinephrine transporters, as well as the sigma 1 and sigma 2 receptors. The affinities of compound **22** for the serotonin, dopamine, and norepinephrine transporters were relatively low with *K*i = 1800, 1053, and 1902 nM, respectively. In addition to these monoamine transporters, compound **22** was found to have moderate affinity for the sigma 1 and 2 receptors with *K*i = 276 and 412 nM, respectively.

**Table 2:** Compound **22** binding studies from PDSP: secondary screen for compound **22** and subsequent *K*i values (list of compound **22** potential targets).

Target	Ki (nM) Compound 22
Sigma 1 receptor	276
Sigma 2 receptor	412
Dopamine Transporter	1053.5
Serotonin Transporter	1800
Norepinephrine Transporter	1902


Since the PDSP screen showed that compound **22** was able to bind to the dopamine transporter (*K*i = 1053 nM), which is also the drug target of amphetamine and cocaine, we hypothesized that the psychostimulant potentiating effects of compound **22** could be mediated by modulation of the dopamine transporter. Therefore, we carried out experiments to directly assess the ability of compound **22** to modulate dopamine transporter activity.

**FIGURE 8:**
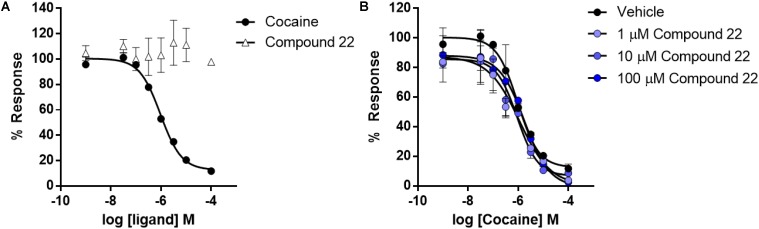
Dose response curves for cocaine and compound **22** effects on dopamine transporter uptake activity. Dose response curves were generated for compound **22** or cocaine by the addition of a range of doses to HEK293 cells stably expressing human HA-DAT. **(A)** Dose response of cocaine and compound **22** on inhibition of uptake; **(B)** cocaine co-treatment with: vehicle, 1, 10, or 100 μM of compound **22**. Error bars represent standard error of mean at *N* = 3.

Cells stably expressing the human dopamine transporter were pre-treated with increasing doses of compound **22** or cocaine which was used as a positive control. The ability of compound **22** to directly disrupt uptake activity was assessed with a fluorescent uptake assay. As shown in **Figure 7**, cocaine dose-dependently inhibited dopamine uptake by DAT (IC_50_ = 0.95 ± 0.02 μM), while compound **22** had no effect on dopamine uptake activity (**Figure 8A**). Next, we assessed whether compound **22** could modulate cocaine inhibitory effects on DAT. As shown in **Figure 7B**, compound **22** at three doses (1, 10, and 100 μM) did not alter the cocaine dose response inhibition of dopamine uptake (**Figure 8B**). These data are in contrast to the results obtained from PDSP, and indicate that if compound **22** binds to the dopamine transporter, it does not block dopamine uptake via the transporter.

## Discussion

In our previous study, compound **22** appeared to be a potential weak antagonist (IC_50_ > 100 μM) for TAAR1 (Lam et al., 2015b). The favorable predicted chemical properties of compound **22** (**Table 1**) led to the hypothesis that compound **22** would cross BBB and have effects *in vivo*. Indeed, our experiments in this study confirmed that compound **22** was able to cross BBB and potentiate the locomotor stimulating effects and stereotypic counts when co-injected with amphetamine and cocaine in WT animals.

Since TAAR1-KO mice have a potentiated locomotor response to amphetamine and cocaine when compared to WT mice (Wolinsky et al., 2007; Lindemann et al., 2008; Di Cara et al., 2011), we hypothesized that the locomotor and stereotypic effects of compound **22** seen in WT mice were consistent with TAAR1-based antagonism. However, we observed that compound **22** could also potentiate cocaine- and amphetamine-mediated locomotor activity and stereotypic counts in TAAR1-KO mice, showing that the *in vivo* locomotor effects of compound **22** are, in fact, TAAR1-independent. Further attempts at elucidating the compound **22** mechanism of action using PDSP suggested that the effects of compound **22** could be mediated by the dopamine transporter. However, our follow up experiments in heterologous cells excluded this possibility. In addition, PDSP also showed the sigma 1 receptor as a hit for compound **22**. The sigma 1 receptor is a single transmembrane protein that is primarily localized in the endoplasmic reticulum. The sigma 1 receptor is expressed in peripheral tissues (Stone et al., 2006), as well as highly expressed in the brain (Alonso et al., 2000; Hayashi and Su, 2005). Previous studies with sigma 1 ligands showed that sigma 1 agonists potentiated cocaine mediated locomotor activity (Menkel et al., 1991; Matsumoto et al., 2001; Rodvelt et al., 2011a,b; Lever et al., 2014; Hong et al., 2017), however, agonists inhibited amphetamine mediated locomotor activity (Poncelet et al., 1993; Rückert and Schmidt, 1993; Guitart et al., 1998; Skuza and Rogóz, 2006). Therefore, it is unlikely that compound **22** is a sigma 1 ligand as we have shown that compound **22** potentiates both cocaine and amphetamine mediated locomotor activity. In sum, our data suggest that compound **22**, which is a low potency TAAR1 antagonist, is able to enhance amphetamine- and cocaine-mediated locomotor activity through a currently unknown mechanism.

Within the basal ganglia circuitry, there are multiple receptor systems that could explain the *in vivo* results we observed with compound **22**. While it is rare for a compound to potentiate both amphetamine- and cocaine-induced locomotor activity and not stimulate locomotor activity alone, several other compounds have been previously discovered that act in a similar mode. In general, such compounds fit into three distinct mechanisms of action: 1) enhanced firing rate of dopaminergic neurons and 2) enhanced stimulation of D2 dopamine receptor expressing medium spiny neurons (MSN).

Given that compound **22** was found to enhance the firing rate of dopamine neurons, we hypothesize the target of compound **22** to be critical for modulating the firing rate of dopamine neurons. There are several presynaptic receptors that regulate this phenomenon. Such receptors include, but are not limited to, the D2 dopamine receptor, TAAR1, and 5HT_2C_ receptors. For example, the 5HT_2C_ receptor antagonist SB232082 potentiates the locomotor stimulating effects of MDMA, amphetamine, fenfluramine, cocaine, methylphenidate, nicotine, and morphine (Fletcher et al., 2006). Mechanistically, the antagonism of the 5HT_2C_ receptors increases the firing rate of dopaminergic neurons in the VTA, resulting in enhanced dopamine release (Millan et al., 1998; Di Giovanni et al., 1999; Di Matteo et al., 1999, 2002). However, it is unlikely that compound **22** is acting as a 5HT_2C_ receptor antagonist since this receptor was not a hit in the PDSP screen. It is possible that compound **22** could bind to another pre or postsynaptic receptor to enhance the firing rate of dopaminergic neurons as a mechanism of action.

Lastly, regulation of locomotor activity can also occur at the level of postsynaptic MSN expressing the dopamine D2 receptor. One potential target of compound **22** could be the adenosine A_2A_ receptor, which is expressed in D2-expressing MSN (Schiffmann et al., 2007; Fredholm et al., 2011). Mechanistically, it has been shown that the A_2A_ receptor has mutual antagonistic activities with the D2 dopamine receptor. Both the A_2A_ receptor and the D2 dopamine receptor have been shown to dimerize *in vitro*, as well as in striatal membrane preparations from rats (Ferre et al., 1991; Yang et al., 1995; Dasgupta et al., 1996; Kamiya et al., 2003). The activation of the A_2A_ receptor via A_2A_ agonists inhibits amphetamine- and cocaine-mediated behaviors (Turgeon et al., 1996; Ferré et al., 1997; Rimondini et al., 1997; Baldo et al., 1999; Chen et al., 2001; Knapp et al., 2001; Filip et al., 2006). Conversely, antagonism of the A_2A_ receptor potentiates amphetamine- and cocaine-mediated behaviors (Casas et al., 1989; Turgeon et al., 1996; Ferré et al., 1997; Fredholm et al., 1999; Shiozaki et al., 1999). Since the adenosine A_2A_ receptor was not tested in the original PDSP screen, it is possible that compound **22** is acting as an adenosine A_2A_ receptor antagonist.

One of the limitations of our study is the species differences between the receptors used for our *in vivo* and *in vitro* experiments. For instance, the original TAAR1 homology model used to identify compound **22** was generated using the human TAAR1 primary amino sequence (Lam et al., 2015b). In addition, the PDSP assays were performed on human proteins. However, our *in vivo* studies were carried out in mice. While the human and mouse TAAR1 receptors share 76% sequence homology (Miller et al., 2005), there are important differences in the affinities for known compounds between these receptors from humans and mice. For instance, EPPTB has a 0.9 nM affinity for the mouse TAAR1; while EPPTB does not bind to the human TAAR1 receptor (Bradaia et al., 2009). Therefore, it is possible that compound **22** could be more selective for a mouse receptor (over a human receptor) that would explain mechanistically the *in vivo* results. Despite species differences, compound **22** still elicits a potentiation of psychoactive-induced locomotor responses in the absence of TAAR1 (TAAR1-KO mice).

## Conclusion

In conclusion, our *in vivo* studies have shown compound **22** to potentiate the locomotor stimulating effects of both amphetamine and cocaine. Our original hypothesis was that compound **22** mediated these effects through the antagonism of TAAR1. However, these findings were also observed in the TAAR1-KO mice, suggesting that compound **22** is not mediating potentiation of amphetamine- and cocaine-induced locomotor response through TAAR1. In collaboration with PDSP, we attempted to determine the target for compound **22**; however, the target for compound **22** remains unknown. Therefore, compound **22** appears to be a potent modulator of dopamine signaling within the brain, through a yet unknown mechanism.

## Author Contributions

VL, AR, AS, SE, IS, and RG contributed conception and design of the study. VL, CM, PB, and WH designed and contributed to experimental data found in the figures of the manuscript. CB and SB contributed to electrophysiology experiments in **Figure 4**. VL wrote the first draft of the manuscript. All authors contributed to manuscript revision, read, and approved the submitted version.

## Conflict of Interest Statement

The authors declare that the research was conducted in the absence of any commercial or financial relationships that could be construed as a potential conflict of interest. The handling Editor declared a shared affiliation, though no other collaboration, with one of the authors PB.

## Supplementary Material

FIGURE S1 Stereotypic counts of C57BL/6J mice co-injected with compound **22** and cocaine. **(A)** Wild type C57BL/6J mice were first habituated for 30 min followed by the co-injection of amphetamine (2 mg/kg) and saline or compound **22** at doses 5, 15, 20, 25, 30, and 50 mg/kg. The locomotor activity was assessed for 60 min after the injection. Sum of stereotypic counts assessed over 60 min after the injection of amphetamine and compound **22**. Data are means ± SEM; *N* = 12–18 for compound **22** treated alone and *N* = 66 for amphetamine treated alone. A one way ANOVA was performed [*F*(5,126) = 4.482, *p* = 0.0009] followed by Dunnett’s *post hoc* analyses (^∗^*p* < 0.05, ^∗∗∗^*p* < 0.001). **(B)** Wild type C57BL/6J mice were first habituated for 30 min followed by co-injection of cocaine (10 mg/kg) and saline or compound **22** at doses 5, 15, and 25 mg/kg. The locomotor activity was assessed for 60 min after the injection. Sum of stereotypic counts assessed over 60 min after injection of cocaine and compound **22**. Data are means ± SEM; *N* = 7–9. One way ANOVA was performed [*F*(3,29) = 4.365, *p* = 0.0118] followed by Dunnett’s *post hoc* analyses (^∗^*p* < 0.05, ^∗∗∗^*p* < 0.001).

FIGURE S2 *In vivo* studies with TAAR1-KO mice and compound **22** on stereotypic counts activity. **(A)** TAAR1-KO mice and their WT littermates (C57BL/6J x 129S2/Sv) were first habituated for 30 min followed by co-injection of amphetamine (2 mg/kg) and saline or compound **22** at doses 2.5, 5, and 15 mg/kg. The sum of stereotypic counts was assessed over 60 min after the injection of amphetamine and compound **22** in WT (solid bars) or TAAR1-KO mice (spotted bars). Data are means ± SEM; *N* = 7–23. One-way ANOVA was performed for each genotype; WT [*F*(3,43) = 1.843, *p* = 0.1536] and TAAR1-KO [*F*(3,43) = 4.303, *p* = 0.0097] followed by Dunnett’s *post hoc* analyses ^∗∗^*p* < 0.01). **(B)** TAAR1-KO mice and WT littermates (C57BL/6J x 129S2/Sv) were first habituated for 30 min, followed by co-injection of cocaine (10 mg/kg) with saline or compound **22** (5, 15, and 25 mg/kg). The sum of stereotypic counts was assessed over 60 min following the injection in WT (solid bars) or TAAR1-KO mice (dotted bars). Data are means ± SEM; *N* = 7–29. A one-way ANOVA was performed for each genotype; WT [*F*(3,37) = 6.696, *p* = 0.001) and TAAR1-KO [*F*(3,51) = 13.57, *p* < 0.0001] followed by Dunnett’s *post hoc* analyses (^∗∗^*p* < 0.01, ^∗∗∗^*p* < 0.001, ^∗∗∗∗^*p* < 0.0001).

TABLE S1 Compound **22** binding studies from PDSP: primary screen for compound **22** and subsequent hits (50% cut-off, hits highlighted in red). The primary screen was performed by PDSP as described previously (Besnard et al., 2012).
