# Supplementary figures and images for: Behavioral Effects of a Potential Novel TAAR1 Antagonist
Source: Front Pharmacol. 2018 Sep 4;9:953. doi: 10.3389/fphar.2018.00953 (PMC6131539; doi:10.3389/fphar.2018.00953)

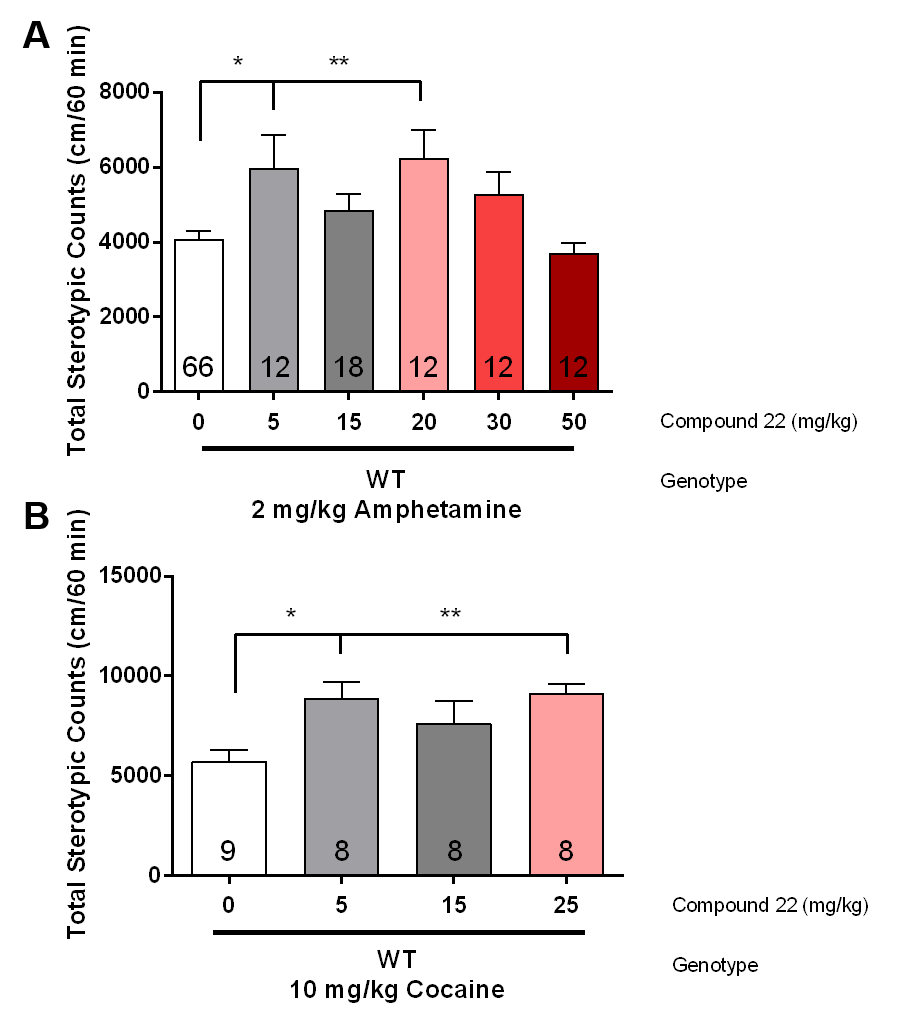

Supplement: FIGURE S1 — Stereotypic counts of C57BL/6J mice co-injected with compound 22 and cocaine. (A) Wild type C57BL/6J mice were first habituated for 30 min followed by the co-injection of amphetamine (2 mg/kg) and saline or compound 22 at doses 5, 15, 20, 25, 30, and 50 mg/kg. The locomotor activity was assessed for 60 min after the injection. Sum of stereotypic counts assessed over 60 min after the injection of amphetamine and compound 22. Data are means ± SEM; N = 12–18 for compound 22 treated alone and N = 66 for amphetamine treated alone. A one way ANOVA was performed [F(5,126) = 4.482, p = 0.0009] followed by Dunnett’s post hoc analyses (∗p < 0.05, ∗∗∗p < 0.001). (B) Wild type C57BL/6J mice were first habituated for 30 min followed by co-injection of cocaine (10 mg/kg) and saline or compound 22 at doses 5, 15, and 25 mg/kg. The locomotor activity was assessed for 60 min after the injection. Sum of stereotypic counts assessed over 60 min after injection of cocaine and compound 22. Data are means ± SEM; N = 7–9. One way ANOVA was performed [F(3,29) = 4.365, p = 0.0118] followed by Dunnett’s post hoc analyses (∗p < 0.05, ∗∗∗p < 0.001). [file Image_1.TIF]

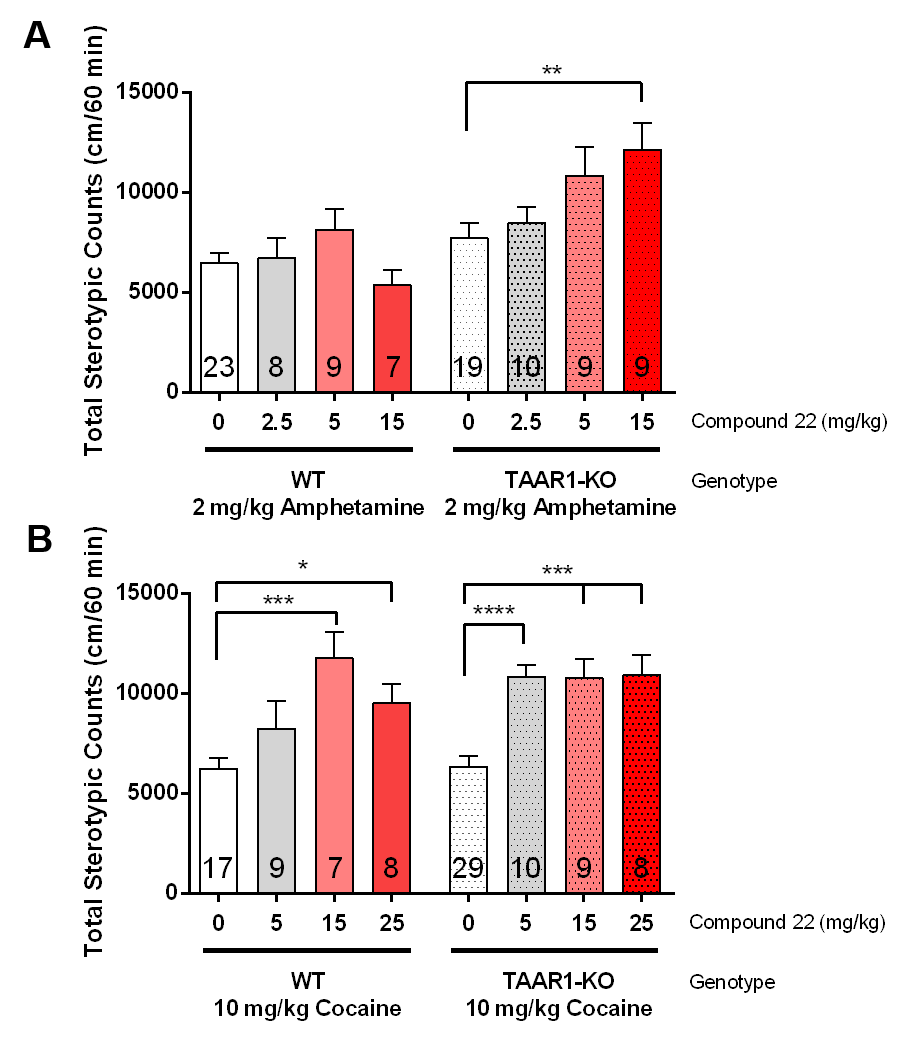

Supplement: FIGURE S2 — In vivo studies with TAAR1-KO mice and compound 22 on stereotypic counts activity. (A) TAAR1-KO mice and their WT littermates (C57BL/6J x 129S2/Sv) were first habituated for 30 min followed by co-injection of amphetamine (2 mg/kg) and saline or compound 22 at doses 2.5, 5, and 15 mg/kg. The sum of stereotypic counts was assessed over 60 min after the injection of amphetamine and compound 22 in WT (solid bars) or TAAR1-KO mice (spotted bars). Data are means ± SEM; N = 7–23. One-way ANOVA was performed for each genotype; WT [F(3,43) = 1.843, p = 0.1536] and TAAR1-KO [F(3,43) = 4.303, p = 0.0097] followed by Dunnett’s post hoc analyses ∗∗p < 0.01). (B) TAAR1-KO mice and WT littermates (C57BL/6J x 129S2/Sv) were first habituated for 30 min, followed by co-injection of cocaine (10 mg/kg) with saline or compound 22 (5, 15, and 25 mg/kg). The sum of stereotypic counts was assessed over 60 min following the injection in WT (solid bars) or TAAR1-KO mice (dotted bars). Data are means ± SEM; N = 7–29. A one-way ANOVA was performed for each genotype; WT [F(3,37) = 6.696, p = 0.001) and TAAR1-KO [F(3,51) = 13.57, p < 0.0001] followed by Dunnett’s post hoc analyses (∗∗p < 0.01, ∗∗∗p < 0.001, ∗∗∗∗p < 0.0001). [file Image_2.TIF]
