# Supplementary material for: Behavioral Effects of a Potential Novel TAAR1 Antagonist
Source: Front Pharmacol. 2018 Sep 4;9:953. doi: 10.3389/fphar.2018.00953 (PMC6131539; doi:10.3389/fphar.2018.00953)
Supplement: TABLE S1 — Compound 22 binding studies from PDSP: primary screen for compound 22 and subsequent hits (50% cut-off, hits highlighted in red). The primary screen was performed by PDSP as described previously (Besnard et al., 2012). [file Table_1.docx]

Supplemental Table 1. Compound **22** binding studies from PDSP: primary screen for compound **22** and subsequent hits (50% cut-off, hits highlighted in red). The primary screen was performed by PDSP as described previously (Besnard et al., 2012).

| **Target** | **% Inhibition** | **Target** | **% Inhibition** |
| --- | --- | --- | --- |
| **5-HT1A** | 9.3 | **D3** | 9.4 |
| **5-HT1B** | -13.5 | **D4** | 17.6 |
| **5-HT1D** | 9.7 | **D5** | -6.8 |
| **5-HT1E** | -1.7 | **DAT** | **60.5** |
| **5-HT2A** | 2.1 | **DOR** | 6.2 |
| **5-HT2B** | 6.7 | **GABAA** | 5.2 |
| **5-HT2C** | 18.5 | **H1** | 6.3 |
| **5-HT3** | 6 | **H2** | 45.2 |
| **5-ht5a** | 15.3 | **H3** | 17.8 |
| **5-HT6** | 4.6 | **H4** | -9.8 |
| **5-HT7** | 20.2 | **KOR** | -10.2 |
| **Alpha1A** | -13.9 | **M1** | 18.3 |
| **Alpha1B** | 12.8 | **M2** | 7.1 |
| **Alpha1D** | 11.6 | **M3** | 15.5 |
| **Alpha2A** | 32.4 | **M4** | 15.9 |
| **Alpha2B** | 22.6 | **M5** | 1.4 |
| **Alpha2C** | -4.3 | **MOR** | -3.7 |
| **AMPA** | 13.4 | **NET** | **93.4** |
| **Beta1** | -3.5 | **NMDA** | -1.7 |
| **Beta2** | -0.6 | **PBR** | 23.1 |
| **Beta3** | -9.1 | **SERT** | **57.3** |
| **BZP site** | 1.1 | **Sigma 1** | **93.2** |
| **D1** | 16.1 | **Sigma 2** | **78.4** |
| **D2** | 36.7 |  |  |
